# Diversity of Plant Communities Surrounding the Hot Springs on the Eastern Flank of the Sierra Madre Oriental, Northeastern Mexico

**DOI:** 10.3390/biology14040382

**Published:** 2025-04-07

**Authors:** Jerjes R. Pantoja-Irys, Edilia de la Rosa-Manzano, José Guadalupe Martínez-Ávalos, Antonio Guerra-Pérez, Arturo Mora-Olivo, Leonardo U. Arellano-Méndez, Rocío Serna-Márquez, Edgar Daniel Salmerón-Carreño, Hugo Mujica-Sánchez

**Affiliations:** 1 Corporación Ambiental de México S.A. de C.V. Texcoco 100, Colonia Satélite Acueducto, Monterrey 64960, Nuevo León, Mexico; jerjes.pantojai@anahuac.mx (J.R.P.-I.); rserna@cam-mx.com (R.S.-M.); esalmeron@cam-mx.com (E.D.S.-C.); mujica@cam-mx.com (H.M.-S.); 2 Instituto de Ecología Aplicada, Universidad Autónoma de Tamaulipas, Avenida División del Golfo Núm, 356, Colonia Libertad, Ciudad Victoria 87019, Tamaulipas, Mexico; jmartin@uat.edu.mx (J.G.M.-Á.); aguerra@uat.edu.mx (A.G.-P.); amorao@uat.edu.mx (A.M.-O.); luarellano@uat.edu.mx (L.U.A.-M.); 3 Facultad de Ingeniería y Ciencias, Universidad Autónoma de Tamaulipas, Ciudad Victoria 87149, Tamaulipas, Mexico; 4 Facultad de Derecho y Ciencias Sociales-Victoria, Centro Universitario Victoria, Ciudad Victoria 87149, Tamaulipas, Mexico

**Keywords:** diversity, *Helietta parvifolia*, plant species conservation, rosetophyll scrub, tropical dry forest

## Abstract

Geothermally heated ecosystems experience extreme temperatures and play important ecological, cultural, and socio-economic roles in many environments. They support a diversity of plants that are often used by local communities. This study focused on five hot springs with varying geochemical traits in the northeast of Mexico. The diversity surrounding each hot spring was analyzed along with the impact of climatic factors. In total, we observed 2022 individual plants across 155 species and 55 families. Shrub strata dominated the rosetophyll scrub and low thorn forest, while tree strata were more common in the tropical dry forest; the herbaceous stratum exhibited the least diversity across all sites. A decrease in species richness was observed at higher elevations, while species abundance showed an inverse trend, being highest in the rosetophyll scrub. The plant communities exhibited clear differentiation in species composition with 30 indicator species identified across the five hot springs. Notably, only *Helietta parvifolia* was found in three sites. The variation in plant abundance was linked to elevation and evaporation. Each type of vegetation surrounding the hot springs demonstrates unique characteristics based on plant composition, suggesting that plant diversity may offer significant benefits to the surrounding communities in the northeast of Mexico.

## Simple Summary

Hot springs host diverse plant communities that hold ecological and socio-economic significance. This study examined plant diversity in five hot springs located in northeast Mexico, considering climatic factors. A total of 2022 individual plants, belonging to 155 species across 55 families, were recorded. Shrub strata primarily dominated the rosetophyll scrub and low thorn forest, while trees were more prevalent in tropical dry forests. Herbaceous species were the least abundant across all sites. Species richness tended to decrease with elevation, whereas abundance was highest in higher elevations. Plant communities displayed distinct species compositions, featuring 30 indicator species, of which only *Helietta parvifolia* was found in three sites. Elevation and evaporation were significant factors influencing plant abundance, highlighting the unique characteristics of the vegetation and its potential benefits to local communities.

## 1. Introduction

Geothermal systems are crucial sources of energy extraction and provide various ecosystem services to numerous rural communities, such as recreational services, biodiversity maintenance, nutrient cycling, and others [1,2]. These environments feature a unique combination of salinity, specialized solutes, precipitates, and temperature ranges from 0 to 91 °C [3], which significantly exceed those of surface waters in lakes and streams. While bacterial diversity [4,5] and certain algal groups [6,7,8] in hot springs have been extensively studied, research on the diversity of plants species surrounding these sulfuric aquatic ecosystems remains limited. Many of these plant species are specialized, endemic, or have medicinal properties [9,10,11,12].

Plant diversity is influenced by climatic variables, particularly temperature and precipitation [13,14,15]. The high temperatures of hot springs remain consistent throughout the year, with minimal variation in water temperature over a year [16]. These thermal gradients affect the phenology, physiology, and distribution of plants in the vicinity of the hot springs, directly impacting their life cycles and adaptive capabilities [10]. Moreover, hot springs provide a permanent water source for plants in direct contact with them, and the evaporation from these springs modifies the surrounding environment by reducing water vapor pressure deficits, thus facilitating the distribution of plants around these water bodies. Common plant species found near hot springs include halophytic grasses, bryophytes, succulents, and various herbaceous plants, which have developed tolerance to high mineral concentrations, unusual humidity conditions, and fluctuating temperatures [17,18].

Plants in these stressful environments tend to be smaller, exhibit slow growth rates, have small, needle-shaped leaves, and show intermittent flowering cycles [19]. Some species develop extensive root systems that allow them to extract nutrients from nutrient-poor soils. For instance, when groundwater is near the surface, plant roots can access this water, ensuring a constant supply and reducing reliance on precipitation [20]. This phenomenon is particularly relevant in arid regions, where the survival of phreatophytic plants depends exclusively on their ability to grow deep roots into underground water sources [21,22].

In northeastern Mexico, floristic studies have been conducted in areas with high plant diversity, including both arid and tropical ecosystems [23,24,25]. These studies are vital for developing effective conservation strategies to address direct and indirect anthropogenic impacts, such as increased local and regional groundwater demands, groundwater contamination, habitat modification or loss, and the broader effects of global climate change [11,26]. Understanding plant diversity in hot springs also helps identify indicator species that can reflect changes in environmental quality and spring conditions, facilitating more precise monitoring of these ecosystems health.

**Figure 1:**
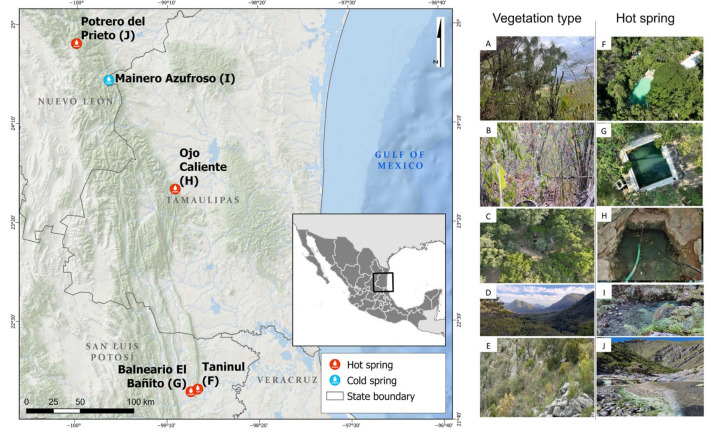
Location of vegetation types surrounding hot springs in northeastern Mexico. Tropical dry forest (**A**,**B**) (Taninul, (**F**) and Bañito, (**G**)), low thorn forest (**C**) (Ojo Caliente, (**H**)), oak forest and submontane scrub (**D**) (Mainero Azufroso, (**I**)), and rosetophyll scrub (**E**) (Potrero del Prieto, (**J**)).

Hot springs in northeastern Mexico emerge from the northeastern flank of the Sierra Madre Oriental, which extends through the states of Nuevo León, Tamaulipas, and San Luis Potosí, Mexico. This region is characterized by a northwest-southeast (NW-SE) trending mountain belt, consisting of elongated and narrow ridges that form the recharge zones of regional aquifers. These aquifers are formed in tropical karst systems of folded and faulted mountains in the south and inactive karst in the north [27], where various hot springs are located, such as Potrero del Prieto, Ojo Caliente, Taninul, El Bañito Spa, and Mainero Azufroso (Figure 1). Although the water temperatures of these springs are similar to those of surrounding streams, they emit hydrogen sulfide vapors, forming unique aquatic ecosystems with green mats and the precipitation of gypsum or calcite nearby. In this study, we aim to (a) analyze plant diversity around five hot springs in northeastern Mexico, (b) compare plant community composition among these sites and identify indicator species, and (c) evaluate the influence of climatic variables on vegetation distribution.

## 2. Materials and Methods

### 2.1. Study Sites

The study was conducted at five locations around hot springs in northeastern Mexico, characterized by low-to-medium enthalpy geothermal activity, hosted in deep and extensive sedimentary basins in northeastern Mexico [28]. These springs are located within the Sierra Madre Oriental, the northeastern plains of Mexico and the coastal plain of the Gulf of Mexico [29]. The well-known Taninul spring (TA) is used for medicinal and recreational purposes, possibly since pre-Hispanic times, and is located on the grounds of a hotel. It is located in the Sierra El Abra-Tanchipa at the foot of the El Abra mountain range. The Balneario El Bañito spring (BA) is currently used for recreational purposes, and is located in the Ciudad Valles municipality, characterized by a topography of gently sloping hills. The Ojo Caliente spring (OC) is situated on private property and serves as a water source for livestock. The Mainero Azufroso spring (MA) emerges from the bed of an intermittent stream in the Sierra La Guitarra, at the foot of the San Manuel Mountain range. Lastly, the Potrero del Prieto spring (PP) is located near the Prieta Linda waterfall and the town of El Potrero del Prieto de Arriba, positioned between the Iturbide anticline and the El Mezquital syncline in the Sierra El Baño, along the bed of the Cabezones River. Table 1 provides a detailed overview of the geological characteristics, climatic conditions, and types of vegetation in these areas.

### 2.2. Data Collected

Three transects, each covering an area of 400 m^2^, were established in different vegetation types near the hot springs, at distances of 30 to 100 m. The vegetation types included tropical dry forest (TDFa, b), low thorn forest (LTF), oak forest–submontane scrub (OF-SMS), and rosetophyll scrub (RS). Within each transect, the tree stratum (species with heights greater than 3 m) was assessed in four 10 × 10 m plots (100 m^2^). The shrub stratum (species with heights greater than one meter, but less than 3 m) was evaluated in four 5 × 5 m plots (25 m^2^), and the herbaceous stratum (species with heights less than or equal to 1 m) was examined in four 1 × 1 m plots (1 m^2^) [30,31,32]. The transects were spaced 50 m apart.

### 2.3. Data Analysis

Diversity among vegetation types. To analyze diversity among vegetation types, we assessed diversity at five sites and generated diversity profiles and multi-assemblage similarity profiles using Hill numbers [33]. We assessed the proportion of the species pool found in each forest type through interpolation and extrapolation analyses [34], which evaluate sample completeness based on sample coverage. This was accomplished using the iNEXT function from the iNEXT package, ver. 3.0.1 [35]. The number equivalents of a known diversity index are the most effective way to quantify diversity [36]. These profiles represent a range of measurements and illustrate diversity as a function of the order q. This parameter controls how sensitive the measurements are to the relative abundance of species. When q = 0, abundance does not factor into the calculation, and diversity reflects species richness alone. When q = 1, the species are weighted according to their relative abundance making them “typical species” in the assemblage (Shannon index). When q = 2, greater weight is assigned to the most abundant species, allowing the measurement to indicate the number of “very abundant species” in the assemblage (as indicated by the Simpson index) [33,37]. For the diversity profiles, the diversity unit represents the effective number of species, while for the multi assemblage similarity profiles, the diversity unit represents the mean overlap of species [38]. To estimate how many species remain undetected in each forest type, we compared the observed species richness to the range of the estimated species richness confidence intervals.

Dissimilarity among vegetation types and factors determining species richness and abundance. Dissimilarity among vegetation types and the factors that determine species richness and abundance were evaluated by nonmetric multidimensional scaling (NMDS). We conducted an ordination based on the abundance data using the Bray distance, with two dimensions and 100 iterations. The ordinations illustrate the distances between transects according to the chosen metric; for this analysis we used the metaMDS function from the vegan package, ver. 2.6–10 [39]. Differences in species composition among vegetation types were evaluated with a multivariate analysis of variance (PERMANOVA) using the adonis function with 999 permutations. Additionally, we applied the Bray–Curtis index to measure biological dissimilarity (or distance) between vegetation types. This index is well established and widely applied, particularly in the assessment of community dissimilarity [40]. For this purpose, we used the vegdist function from the vegan package [39].

Indicator taxa analysis was conducted using the multipatt function from the indicspecies package to assess the significance of indicator values.

Subsequently, a redundancy analysis (RDA) was performed to estimate the total variation in vegetation type data that can be explained by the climatic variables such as precipitation, temperature and evaporation, and elevation. Climatic data for the study sites were sourced from the nearest meteorological stations. Data from each meteorological station were obtained from the Comision Nacional del Agua. Prior to analysis, data were Hellinger-transformed using the decostand function from the vegan package. Hellinger transformation was applied to reduce the influence of large abundances and zero inflation, allowing Euclidean distances to better capture ecological dissimilarities among sites. The RDA was executed using the rda function from the same vegan package [39]. The statistical significance of the canonical axes obtained was determined through permutation tests (*n* = 999) using the anova.cca function. In addition, inertia values were calculated and the results were graphically represented using biplots to illustrate the relationships between the explanatory and response variables. All data analyses were performed using R software version 4.4.2.

## 3. Results

### 3.1. General Floristic Composition and Abundance

**Figure 2:**
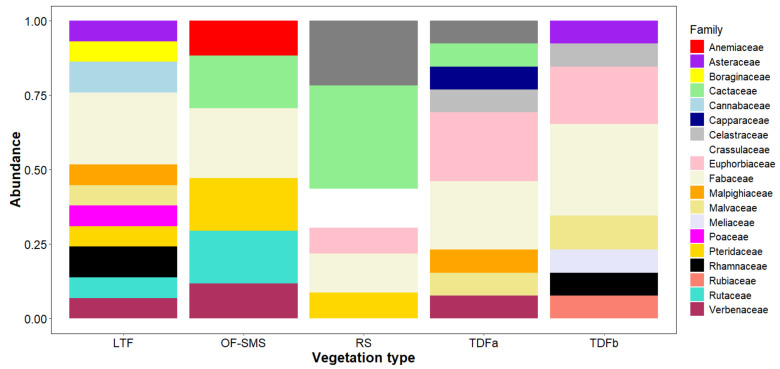
Abundance of plant families found surrounding hot springs on the eastern flank of the Sierra Madre Oriental, northeastern Mexico. Only families with more than two plant species are presented. The vegetation types associated with each hot spring are as follows: low thorn forest (LTF, Ojo Caliente), oak forest and submontane scrub (OF-SMS, Mainero Azufroso), rosetophyll scrub (RS, Potrero del Prieto), and tropical dry forest (TDFa, Taninul and TDFb, Bañito).

A total of 155 taxa, belonging to 131 genera and 55 families, were recorded around the five hot springs studied. Fabaceae was the most represented family, with 28 taxa, accounting for 14.97% of the overall flora. This was followed by Euphorbiaceae which had 15 taxa (8.05%), Cactaceae with 13 taxa (6.95%), and Malvaceae with eight taxa (4.27%). The families with the highest number of genera surrounding each spring were as follows: Fabaceae, which predominated in the tropical deciduous forest (TDFb) of the BA hot spring, in the low thorn forest (LTF) of the OC hot spring and in the oak forest and submontane scrub (OF-SMS) of the MA hot spring. Cactaceae was particularly prominent in the rosetophyll scrub (RS) of the PP hot spring. In the TDFa of the TA hot spring, both Euphorbiaceae and Fabaceae were well represented (Figure 2).

**Figure 3:**
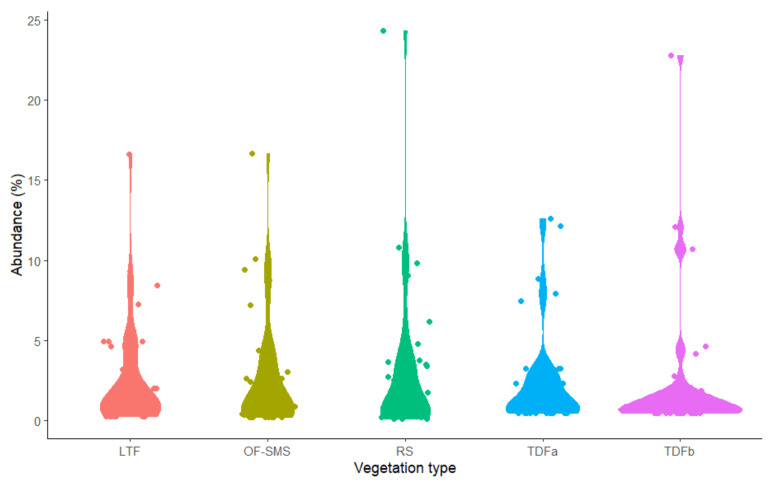
Abundance of plant families surrounding hot springs on the eastern flank of the Sierra Madre Oriental, northeastern Mexico. Only families with more than two plant species are presented. The vegetation types associated with each hot spring are as follows: low thorn forest (LTF, Ojo Caliente), oak forest and submontane scrub (OF-SMS, Mainero Azufroso), rosetophyll scrub (RS, Potrero del Prieto), and tropical dry forest (TDFa, Taninul and TDFb, Bañito).

A total of 2022 individual plants were recorded surrounding the five hot springs. The RS site recorded the highest number with 795 individuals, followed by the OF-SMS with 456, LTF (343), TDFa with 215, and TDFb (214) (Figure 3). The most abundant species by vegetation type were as follows: *Agave lechuguilla* was predominant in the RS, contributing 24.3% (193 individuals); *Guazuma ulmifolia* Lam., made up 12.09% (26 individuals) in the TDFb; *Esenbeckia runyonii* C.V. Morton with 16.6% (76 individuals) in the OF-SMS; *Callaeum macropterum* (Moc. & Sesse ex DC.) D.M. Johnson represented 16.61% (57 individuals) in the LTF; and *Lysiloma divaricatum* J.F.Macbr. comprised 12.61% (27 individuals) in the TDFa.

**Figure 4:**
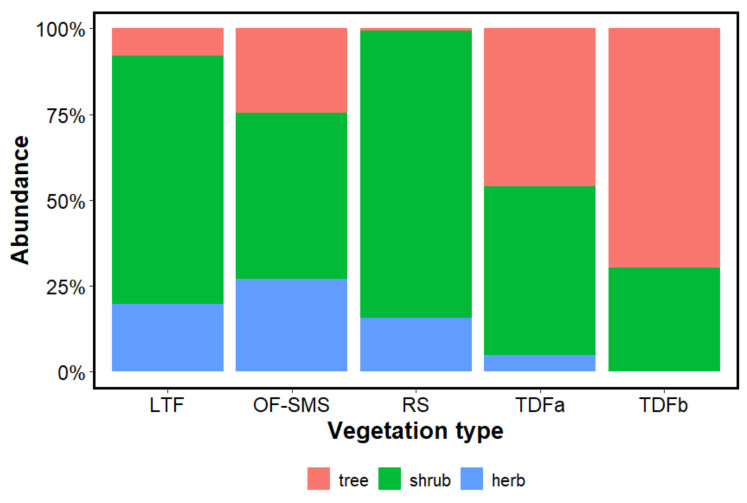
Shows the abundance of plant species surrounding the hot springs on the eastern flank of the Sierra Madre Oriental, northeastern Mexico according to life forms: trees, shrubs, and herbs. The vegetation types associated with each hot spring are as follows: low thorn forest (LTF, Ojo Caliente), oak forest and submontane scrub (OF-SMS, Mainero Azufroso), rosetophyll scrub (RS, Potrero del Prieto), and tropical deciduous forest (TDFa, Taninul and TDFb, Bañito).

When examining the biological forms (tree, shrub, and herbaceous) that prevail around each spring, the shrub strata contributed 83.62% in the RS and 72.30% in the LTF. The tree stratum was prominent in the TDFb accounting for 61.79%; however, in the RS, this component made up only 0.75%, with *Prosopis glandulosa* (L.D. Benson) M.C. Johnst. being the sole tree species present (Figure 4). The herbaceous stratum contributed 27.15% of the species in the OF-SMS, 19.85% in the LTF, 15.61% in the RS, and just 4.81% in the TDFa. The only herbaceous species recorded were *Tradescantia* sp., *Hybanthus mexicanus* Ging., and *Zamia fischeri* Miq. (Figure 4).

### 3.2. Plant Diversity Around the Hot Springs

Sample coverage values for each vegetation type surrounding the hot springs were all above 0.93 (see Figure S1).

**Figure 5:**
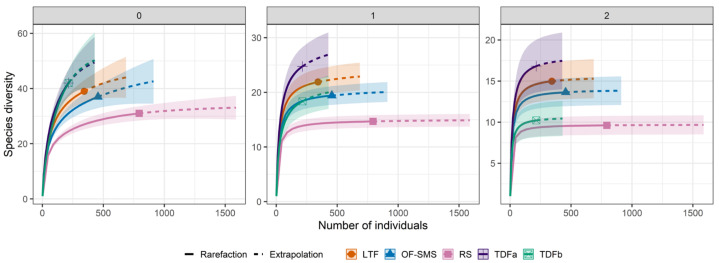
Interpolation and extrapolation curves of the plant species diversity around hot springs in northeastern Mexico. The interpolated (observed) species richness of plants was obtained by merging abundance and species richness data from transects across various vegetation types. Meanwhile, the extrapolated (expected) species richness for each vegetation type was estimated based on the highest maximum of individuals (ca. 1000). The first Hill number (0) highlights species richness, the second Hill number (1) emphasizes the Shannon diversity exponent, and the third Hill number (2) represents the inverse of the Simpson diversity index. Each hot spring is associated with distinct vegetation types: tropical deciduous forest in Taninul (TA), and El Bañito (BA), oak forest and submontane scrub in Mainero Azufroso (MA), low thorn forest in Ojo Caliente (OC), and rosetophyll scrub in Potrero del Prieto (PP). The bands depicted in the figure represent the 95% confidence interval.

The effective species richness (q = 0) did not differ among four of the five vegetation types around the hot springs, as indicated by the overlap of their confidence intervals. The lowest effective species richness was observed at the PP hot spring (Figure 5). Specifically, 42 species were recorded in the TDF of the TA and BA hot springs, 39 species in the thorn forest (LTF) of the OC hot spring, 37 species in the oak forest and submontane scrub (OF-SMS) of the MA hot spring, and 31 species in the rosetophyll scrub (RS) of the PP hot spring.

Species abundance was evenly distributed across species (q = 1), with higher numbers in the TDFa (24.3 species), LTF (20.1 species), TDFb (18.3 species), and OF-SMS (17.3 species) compared to the RS (12.6 species) (Figure 5). For q = 2 (inverse Simpson index), the TDFa (16.6 species), LTF (1.2 species), TDFb (10.2 species), and OF-SMS (12.7 species) did not show any differences among each other, and their species richness was greater than that of the RS (8.8 species) (Figure 5).

Extrapolation of species richness indicated that increasing the sample size (number of individuals per transect) would yield only two additional species in the RS (Table S1), while eight and seven species could be added in TDFa and TDFb, respectively, and four and five species in LTF and OF-SMS, respectively (Table S1).

**Figure 6:**
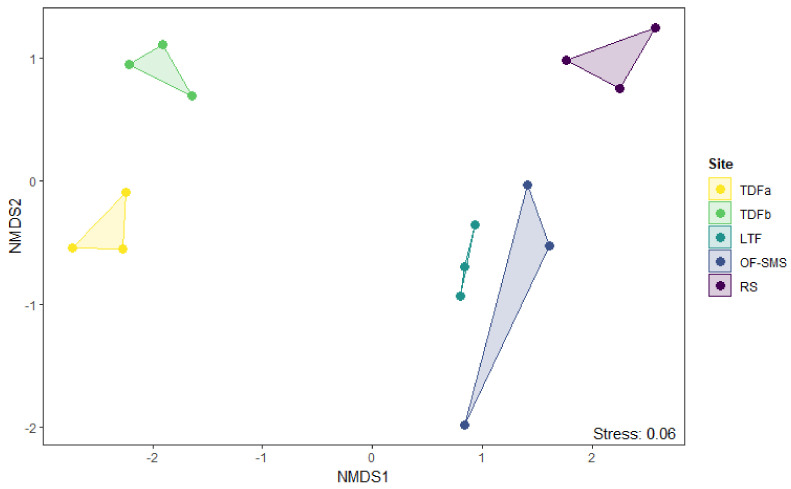
Nonmetric multidimensional scaling (NMDS) ordination of vegetation types found around hot springs, categorized by their abundance. The codes for the different vegetation types are shown on the right legend: tropical deciduous forest (TDFa in Taninul, and TDFb in El Bañito), oak forest and submontane scrub (OF-SMS in Mainero Azufroso), low thorn forest (LTF in Ojo Caliente), and rosetophyll scrub (RS in Potrero del Prieto).

Dissimilarity analyses showed a clear differentiation in the assemblage of vegetation types surrounding the hot springs in terms of taxonomic composition (Figure 6; Table 2). The lowest dissimilarity of 77% was observed between the OF-SMS and LTF sites. In contrast, comparisons between vegetation types at the other sites exceeded 89% (Table 2). A total of 30 indicator plant species were identified across the five vegetation types (*p* < 0.05; Table S3). Of these, 25 species were unique indicators to a single site, four species were found in two sites, and only *Helietta parvifolia* A. Gray (Benth) was present in three sites: OF-SMS, LTF, and RS.

The ordination using nonmetric multidimensional scaling (NMDS) produced a two-dimensional solution with a stress of 0.06 at 100 iterations. Significant differences in species composition were observed among the various vegetation types, as indicated by the Adonis, R^2^ = 0.67, F_4,14_ = 5.22, *p* < 0.001. This finding was further supported by the absence of overlap in species composition across all vegetation types. In the ordination space, the TDF around the two hot springs is positioned at the left end corresponding to low-altitude sites. In contrast, the OF-SMS and the LTF are located at the lower right end of the ordination, which are located at a medium altitude. Finally, the RS is located at the upper right end, which corresponds to the highest altitude among the studied sites (Figure 6).

### 3.3. Climatic Variables Determining the Compostition of Vegetation Types

**Figure 7:**
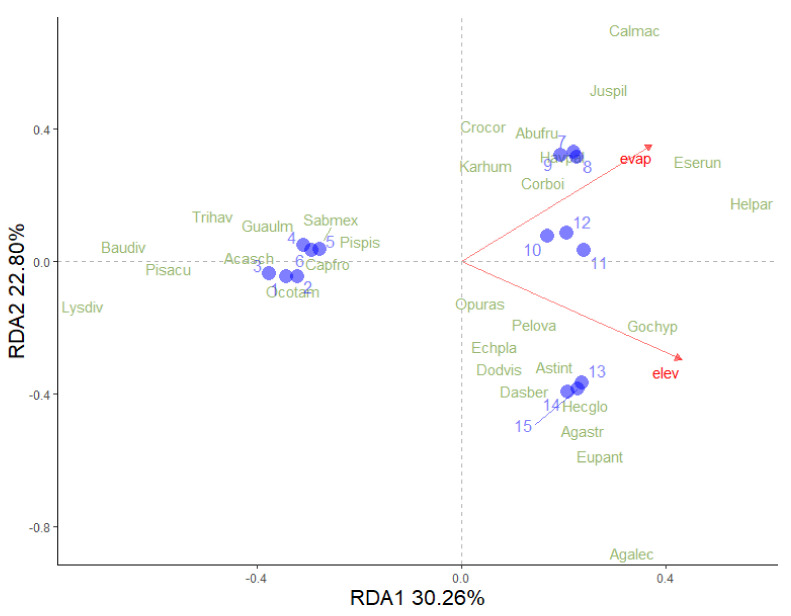
Redundance analysis (RDA) of plant species surrounding each hot spring in northeastern Mexico and climatic variables. The relationship between climatic variables (red arrows) and the distribution of plant species (green letters) and study site (light blue circles) is shown. The vegetation type codes are as follows: tropical deciduous forest around TA (circle 1, 2, and 3), tropical dry forest in BA (circles 4, 5, and 6), low thorn forest (circles 7, 8, and 9), oak forest and submontane scrub (circles 10, 11, and 12), and rosetophyll scrub (circles 13, 14, and 15). The first axis accounts for 30.26% of the total variation, while the second axis accounts for 22.80%. Abbreviations used are evap = evaporation and elev = elevation.

Elevation and evaporation accounted for 53% of the variance in vegetation composition. The first axis explained 30.26% of the variance, while the second axis accounted for 22.80% (Figure 7). High evaporation rates were related to the LTF of the OC hot spring and OF-SMS of the MA hot spring, whereas low evaporation rates were associated with TDF of the TA and BA hot springs. In contrast, high elevation was linked to the RS of the PP hot spring, although there was a low correlation between these two variables. Species such as *Lysiloma divaricatum*, *Bauhinia divaricata* L., *Pisonia aculeata* L., and *Trichilia havanensis* Jacq. (from TDF) were found in areas with low levels of evaporation and elevation. In contrast, species such as H. *parvifolia*, *Esenbeckia runyonii* C.V. Morton, *Agave lechuguilla* Torr., *Gochnatia hypoleuca* (DC.) A. Gray, *Agave striata* Zucc., and *Euphorbia antisyphilitica* Zucc. (from the RS) were associated with higher values of elevation and evaporation.

## 4. Discussion

### 4.1. Floristic Composition and Abundance

The hot springs of northeastern Mexico, located on the eastern flank of the Sierra Madre Oriental, are surrounded by diverse vegetation types shaped by altitudinal gradients. These range from tropical deciduous forests at lower elevations to oak forests and rosetophyll scrub in higher mountainous regions. A total of 155 plant species were identified across the study site, which is comparable to the Taupō Volcanic Zone geothermal system in New Zealand, where 16 vegetation types were described within a similar area [41]. However, this number is only representative of about one-third of the 444 species reported in the Canadian hot springs [42]. The greater floristic richness in the Canadian study may be attributed to the inclusion of both aquatic and terrestrial species, increasing the total number of recorded species across different vegetation strata.

Some groups, such as mosses and hornworts, were absent in this study, probably due to recreational and tourism impacts at tropical sites like Taninul and El Bañito. Anthropogenic disturbance may affect the bryophyte population and other sensitive vegetation [18]. Moreover, seasonal variability likely influenced the occurrence of herbaceous species, which tend to fluctuate significantly with growing season [43].

Fabaceae was the most prevalent family in four of the five vegetation types, consistent with its dominance in lowland forests and scrublands throughout northeastern Mexico [31,44], and other regions of the country [45,46,47,48]. In contrast, the highest diversity of Cactaceae occurred in the rosetophyll scrub of the PP hot spring, reflecting the morphological and physiological adaptations of cacti to arid environments with low rainfall and high temperatures [49]. This aligns with findings from arid zones in southeastern Coahuila, Mexico where up to 30 cacti species have been reported [50].

In tropical deciduous forest, the lower abundance of tree species such as *E*. *runyonii*, *G*. *ulmifolia*, *Savia sessiliflora* (Sw.) Willd., and *L*. *divaricatum* has been observed [23]. This reduced abundance may reflect biological characteristics, including low population density. Notably, *P*. *glandulosa* was the only tree species found in the xerophytic scrubland of the PP hot spring. This species demonstrates remarkable adaptability, developing a deep root system that can reach depths up to 50 m and extend laterally up to 15 m, allowing it to access groundwater and thrive in arid conditions [51]. This highlights the ecological role that hot springs may play as critical water sources in semiarid ecosystem.

Shrub dominated the vegetation structure across most sites, indicating that trees and herbaceous layers were less prominent. In tropical deciduous and low thorn forest, dense canopy cover may reduce light availability, limiting herbaceous growth. Under such conditions, species with strong edaphic adaptations may thrive under shaded conditions where competition for light is minimized [52,53].

In the herbaceous stratum in the tropical deciduous forest at Taninul hot spring, *Zamia fischeri* was recorded, a cycad endemic to Mexico and listed as Endangered under both national legislation (NOM-059-SEMARNAT, 2010) and the International Union for Conservation of Nature (IUCN) Red List [54]. This species is under severe threat due to habitat loss and illegal collection. Similarly, in the rosetophyll scrub of PP hot spring we identified some endemic species such as *Dasylirion berlandieri* S. Watson, *Echinocactus platyacanthus* Link & Otto, and *Thelocactus conothelos* (Regel & Klein bis) F.M. Knuth. Several of these species are categorized as Endangered or Critically Endangered by the IUCN [54] due to their restricted distributions and overexploitation for ornamental trade. The conservation of vegetation surrounding Taninul and Potrero del Prieto is vital for ensuring the survival of these and other threatened species such as *Beaucarnea inermis* (S. Watson) Rose. This finding highlights the urgent need to implement site-specific management plans and habitat protection strategies that prioritize endemic and at-risk plant taxa. More broadly, conserving these ecosystems contributes to global diversity targets and ensures the continuity of key ecological functions.

### 4.2. Diversity Patterns Across Vegetation Types Around the Hot Springs

Based on species richness, the tropical deciduous forests surrounding the TA and BA hot springs exhibited the highest diversity, followed by the oak forest ecotone with submontane scrub, while the rosetophyll scrub showed the lowest diversity. This result support the general pattern of those tropical environments—characterized by higher precipitation, lower elevation, and southern latitudes—harbor greater plant diversity than more northern sites with higher altitudes, lower temperatures, and precipitation, such as the rosetophyll scrub of PP hot spring.

The species richness in the tropical deciduous forests is comparable to that reported for lowland forests of Campeche, Mexico, where similar richness values have been observed among tree species alone [46]. However, deciduous forests in southern Mexico often exhibit three times greater richness [45]. Further sampling in the tropical deciduous forests around TA and BA could yield an estimated seven to eight additional species, approaching the species accumulation curve’s asymptote, this expected richness would still be low compared to the more southerly deciduous forests of Mexico [47,55] and even below the national average for seasonally dry tropical forests (58 species) [56].

Tree diversity in tropical forests is primarily driven by annual precipitation and seasonality patterns [57,58,59]. However, in dry forests with varying rainfall regimes, precipitation–diversity relationships may be more complex and site specific [56]. Additional drivers such as soil type, topography, and species dispersal limitations must be considered when explaining spatial variation in richness around the hot springs [60,61].

Species evenness and dominance were generally similar across four sites, with lower values in the rosetophyll scrub at PP hot spring. At this site, *Agave lechuguilla* Torr. was the most abundant and dominant species, functioning as a clear ecological indicator (Table S3). This species is widely prevalent in arid regions of Mexico due to its strong capacity for vegetative reproduction, forming dense populations in arid environments [62]. Rosetophyll scrublands exhibit low diversity and low dominance due to the extreme climatic and edaphic conditions that few species can tolerate. In contrast, tropical forests usually exhibit higher dominance of shrubs and trees [63].

### 4.3. Floristic Dissimilarity and Ecological Connectivity

The dissimilarity analyses revealed that the plant communities associated with hot springs exhibit a heterogeneous floristic composition. The lowest dissimilarity values occurred between the oak forest–submontane scrub (OF-SMS) and the low thorn forest (LTF), likely due to the presence of shared species adapted to intermediate elevations and ecotonal conditions. Species like *Acacia berlandieri* Benth. and *H. parvifolia*, typical of submontane scrublands, also appear in lowland forests and or integrate with other types of scrublands [64].

Among these species, *H. parvifolia* was present in three vegetation types (LTF, OF-SMS, and RS) and identified as an indicator species. Its presence in diverse ecosystems suggests a broad ecological tolerance and adaptability to different microclimatic and edaphic conditions [64,65]. Furthermore, this species displays some degree of shade tolerance, which allows it to establish itself under the canopy of mature vegetation in both flat areas and submontane zones at elevations of up to 800 m [65,66]. The distribution of *H*. *parvifolia* supports the idea that certain floristic elements function as ecological connectors across contrasting habitats, promoting continuity between vegetation types. Environmental heterogeneity around the hot spring enables the coexistence of species with diverse ecological strategies, enhancing overall floristic and ecosystem differentiation.

### 4.4. Climatic Variables Determining the Abundance and Composition of the Vegetation Types

The redundancy analysis, RDA, indicated that climatic factors play a crucial role in determining species distribution and abundance [67]. Elevation was closely associated with the rosetophyll scrub at the PP hot spring, where species richness was lowest. This relationship aligns with general observations that species richness tends to decrease gradually and continuously with increasing elevation [68]. However, some studies suggest a mid-elevation peak in richness due to optimal environments conditions [68,69,70,71,72]. Diversity changes along elevation gradients result from complex interactions among abiotic factors (e.g., wind, cloud cover, temperature, topography, soil nutrients, and drainage) and biotic factors (e.g., pollinators, seed dispersers, herbivores, and parasites), all of which influence reproductive success and species establishment [57,73,74]. A more detailed analysis of the interactions between these factors would provide a deeper understanding of the structure and dynamics of these ecosystems.

Evaporation was positively associated with the low thorn forest of the OC hot spring as well as the oak forest and submontane scrub of the MA hot springs. These sites are located at middle elevation with moderate precipitation levels (Table 1). At the MA hot spring, local hydrological features such as stream formation, tree cover, and spring proximity may contribute to increased evaporation rates. This relationship has been documented in studies addressing interaction among precipitation, canopy cover, and potential evaporation [75]. The low correlation between elevation and evaporation in the present analysis suggests these variables may have an independent effect on vegetation or may be mediated by other environment factors not considered, such as soil temperature. This underscores the need for further research to assess the influence of additional variables on the structure and composition of vegetation surrounding hot springs.

## 5. Conclusions

Plant communities that develop around hot springs in the Sierra Madre Oriental of Mexico are diverse and heterogeneous. A decline in diversity was observed with increasing elevation, while species abundance showed an opposite trend, suggesting that elevation acts as a filter selecting for species that are adapted to more extreme conditions. Each community contributes significantly to the conservation of biodiversity by hosting unique species assemblages, including endemics and species under risk categories. Protecting these communities is essential to maintain ecological functionality and avoid the loss of floristic elements of high ecological value.

This study establishes a baseline for future conservation-oriented research on ecosystems associated with hot springs. Local degradation in the study area is already evident through deforestation of surrounding vegetation, unregulated tourism activities, and unsustainable water extraction, which collectively alter habitat structure and hydrological regimes [76]. These pressures, if unaddressed, may accelerate landscape fragmentation and compromise key ecosystem services such as nutrient regulation, biomass storage, and biodiversity support. Therefore, it is imperative to strengthen management and conservation strategies to mitigate the impacts of environmental and anthropogenic pressures on these ecosystems.

## Figures and Tables

**Table 1:** Types of vegetation surrounding the hot springs located on the eastern flank of the Sierra Madre Oriental of Mexico, and the geological and climatic traits.

Vegetation Type	Hot Spring	Formation	Elevation (m)	Average Annual Temperature (°C)	Annual Precipitation (mm)
Tropical deciduous forest	Taninul (TA)	El Abra	37	25	1165.4
Tropical deciduous forest	El Bañito (EB)	San Felipe	164	24.5	1224.4
Low thorn forest	Ojo Caliente (OC)	San Felipe	340	24.5	743
Oak forest–submontane scrub	Mainero Azufroso (MA)	Taraises	492	21.3	993.8
Rosetophyll scrub	Potrero del Prieto (PP)	Lower Tamaulipas	1609	18.2	361.8

**Table 2:** Dissimilarity (Bray–Curtis) of the vegetation types surrounding hot springs on the eastern flank of the Sierra Madre Oriental, northeastern Mexico. The vegetation types associated with each spring are as follows: tropical deciduous forest in the Taninul (TDFa) and the Bañito (TDFb), low thorn forest (LTF) in the Ojo Caliente, oak forest and submontane scrub (OF-SMS) in the Mainero Azufroso, and rosetophyll scrub (RS) in the Potrero del Prieto.

	TDFa	TDFb	LTF	OF-SMS
TDFb	0.8927			
LTF	0.9856	0.9784		
OF-SMS	0.9910	1.0	0.7797	
RS	1.0	0.9980	0.9384	0.9392

## Data Availability

Data are contained within the article and Supplementary Materials.

## Supplementary Materials

The following supporting information can be downloaded at https://www.mdpi.com/article/10.3390/biology14040382/s1. Figure S1: Sample coverage curves based on the number of individuals across five vegetation types surrounding hot springs on the eastern flank of the Sierra Madre Oriental, northeastern Mexico. The vegetation types associated with each hot spring are as follows: tropical deciduous forest (TDF, Taninul and Bañito), oak forest and submontane scrub (OF-SMS, Mainero Azufroso), low thorn forest (LTF, Ojo Caliente), and rosetophyll scrub (RS, Potrero del Prieto). The bands correspond to the 95% confidence interval. Table S1: Physical–chemical traits of hot springs on the eastern flank of the Sierra Madre Oriental, northeastern Mexico. TA = Taninul; BA = Bañito; OC = Ojo Caliente; MA = Mainero Azufroso; PP = Potrero del Prieto. Temp = temperature; CE = electrical conductivity; ORP = oxidation; reduction power; DO = dissolved oxygen. Measurements were made in March 2023. Table S2: Analysis of plant diversity surrounding the hot springs through interpolation **(**rarefaction) and extrapolation (iNEXT). The vegetation types associated with various hot springs are tropical deciduous forest (TA, Taninul and BA, Bañito), oak forest and submontane scrub (MA, Mainero Azufroso), low thorn forest (OC, Ojo Caliente), and rosetophyll scrub (PP, Potrero del Prieto). The observed rarefaction species richness of the plants was calculated by merging the observed abundance and species richness from individual transects for each vegetation type. In contrast, the extrapolated (expected) species richness for each vegetation type was determined based on the highest maximum number of individuals recorded. The first column represents the “vegetation type” (VT) surrounding each hot spring, while the “row number” m indicates the sample size (abundance, number of individuals). The “method” column specifies whether the species richness was interpolated, observed (total species richness), or extrapolated (based on maximum abundance). The “order” column reflects the value of q, and the columns qD, qD.LCL, and qD.UCL present the estimated species richness and corresponding lower and upper limits. Additionally, the columns SC, SC.LCL, and SC.UCL provide sample coverage estimates ranging from 0 to 1 along with their respective lower and upper limits. Table S3: Indicator species by vegetation type surrounding the hot springs on the eastern flank of the Sierra Madre Oriental, northeastern Mexico, and a combination of those by the IndVal result. Signif. codes: 0 = ***; 0.001 = **; 0.01 = *.
