# Supplementary material for: Diversity of Plant Communities Surrounding the Hot Springs on the Eastern Flank of the Sierra Madre Oriental, Northeastern Mexico
Source: Biology (Basel). 2025 Apr 7;14(4):382. doi: 10.3390/biology14040382 (PMC12025227; doi:10.3390/biology14040382)
Supplement: Supplementary file 1 [file biology-14-00382-s001.zip › Figure S1.pdf]

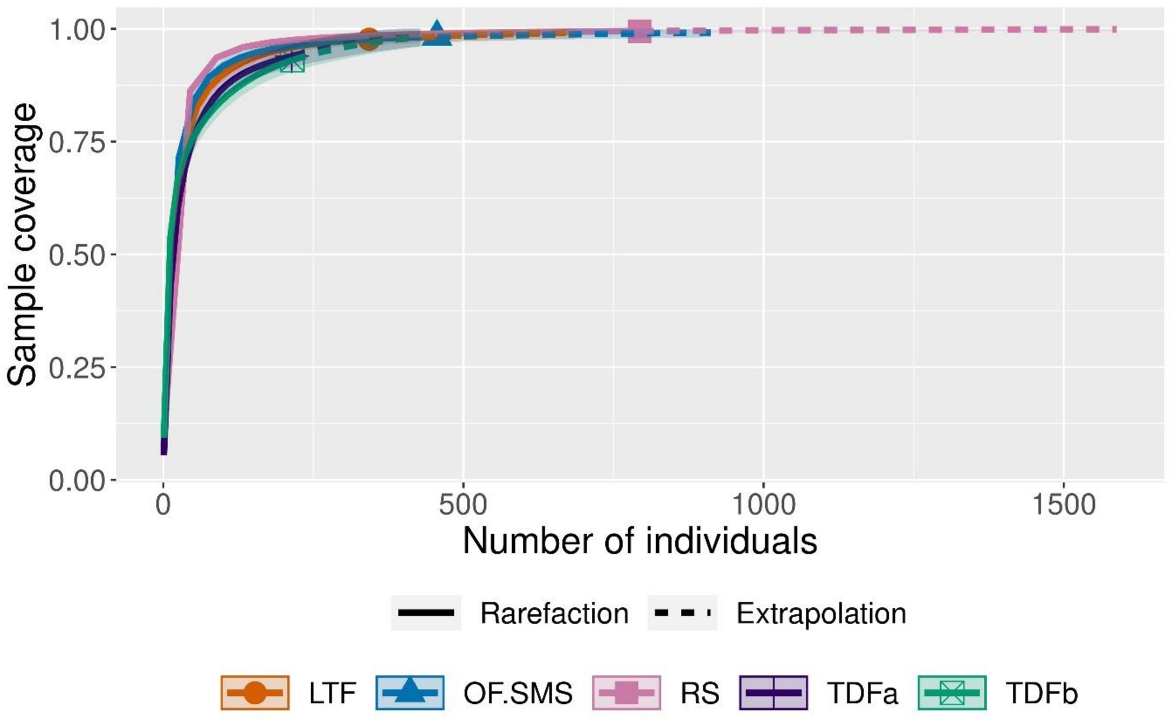

Figure S1 Sample coverage curves based on the number of individuals of five vegetation types around hot springs on the eastern flank of the Sierra Madre Oriental, northeastern Mexico. Vegetation type per hot spring is: tropical deciduous forest (TDFa, Taninul and TDFb, Bañito), oak-forest and submontane scrub (OF-SMS, Mainero Azufroso), low thorn forest (LTF, Ojo Caliente), and rosetophyll scrub (RS, Potrero del Prieto). The bands correspond to the 95% confidence interval.
