# Supplementary material for: Diversity of Plant Communities Surrounding the Hot Springs on the Eastern Flank of the Sierra Madre Oriental, Northeastern Mexico
Source: Biology (Basel). 2025 Apr 7;14(4):382. doi: 10.3390/biology14040382 (PMC12025227; doi:10.3390/biology14040382)
Supplement: Supplementary file 1 [file biology-14-00382-s001.zip › Table S1.pdf]

Table S1. Physical-chemical traits of hot springs on the eastern flank of the Sierra Madre Oriental, northeastern Mexico. TA = Taninul, BA = Bañito, OC = Ojo Caliente, MA = Mainero Azufroso, PP = Potrero del Prieto. Temp = temperature, CE = electrical conductivity, ORP = oxidation, reduction power, DO = dissolved oxygen. Measurements were made in March 2023.

Spring	Temp (ºC)	pH (0-14)	CE (µS/cm)	ORP (mV)	DO (mg/L)	Alkalinity (mg/L)
TA	38.00	6.52	1671	-7.4	0.05	350.63
BA	32.60	7.27	1102	-154.7	1.22	259.63
OC	31.30	6.82	1049	70	1.29	217.35
MA	19.90	7.4	362.1	-150.5	3.56	182.07
PP	26.30	6.94	2421	-247.9	0.81	160.83
