# Supplementary material for: Diversity of Plant Communities Surrounding the Hot Springs on the Eastern Flank of the Sierra Madre Oriental, Northeastern Mexico
Source: Biology (Basel). 2025 Apr 7;14(4):382. doi: 10.3390/biology14040382 (PMC12025227; doi:10.3390/biology14040382)
Supplement: Supplementary file 1 [file biology-14-00382-s001.zip › Table S2.pdf]

Table S2. Interpolation **(**rarefaction) and extrapolation analysis (iNEXT) of plant diversity occurring around hot springs: vegetation type per hot spring is: tropical deciduous forest (TA, Taninul and BA, Bañito), oak-forest and submontane scrub (MA, Mainero Azufroso), low thorn forest (OC, Ojo Caliente), and rosetophyll scrub (PP, Potrero del Prieto). The rarefaction (observed) species richness of plants was obtained by merging the observed abundance and species richness of individual transects per vegetation type. Extrapolated (expected) species richness for each vegetation type was based on the highest maximum number of individuals. The first column indicates the vegetation type (VT) around hot spring, and the row number, m indicates the sample size (abundance, number of individuals), method indicates whether species richness was *interpolated*, observed (total species richness) or *extrapolated* (based on maximum abundance), order indicates the value of q, qD, qD.LCL and qD.UCL indicate estimated species richness and corresponding lower and upper limits, SC, SC.LCL and SC.UCL indicate sample coverage estimate (range: 0-1) and corresponding lower and upper limits.

VT	m	Method	Order.q	qD	qD.LCL	qD.UCL	SC	SC.LCL	SC.UCL
TDFa	1	Rarefaction	0	1	1	1	0.0550656	0.04221586	0.06791533
TDFa	12	Rarefaction	0	9.21581153	8.76151473	9.67010833	0.42909063	0.37498542	0.48319583
TDFa	24	Rarefaction	0	14.9817916	13.9348269	16.0287564	0.60246279	0.55778175	0.64714382
TDFa	36	Rarefaction	0	19.2094568	17.7158825	20.7030311	0.69254244	0.65724555	0.72783934
TDFa	48	Rarefaction	0	22.5623091	20.732718	24.3919001	0.74984141	0.71996655	0.77971627
TDFa	59	Rarefaction	0	25.1149396	23.0381253	27.1917538	0.78774438	0.76075302	0.81473574
TDFa	71	Rarefaction	0	27.4804926	25.175781	29.7852042	0.81921911	0.79418819	0.84425003
TDFa	83	Rarefaction	0	29.5102691	27.0061856	32.0143527	0.84350203	0.81968262	0.86732144
TDFa	95	Rarefaction	0	31.2791728	28.5943712	33.9639745	0.86251434	0.83936937	0.88565931
TDFa	107	Rarefaction	0	32.8427979	29.9890254	35.6965704	0.87756996	0.85469677	0.90044314
TDFa	118	Rarefaction	0	34.1317316	31.1289104	37.1345528	0.8887315	0.86585768	0.91160532
TDFa	130	Rarefaction	0	35.409999	32.2471572	38.5728408	0.89873159	0.87568623	0.92177694
TDFa	142	Rarefaction	0	36.5781343	33.2549488	39.9013199	0.90703019	0.88371834	0.93034205
TDFa	154	Rarefaction	0	37.6539534	34.1678552	41.1400516	0.91407801	0.89046285	0.93769317
TDFa	165	Rarefaction	0	38.5702331	34.9312497	42.2092166	0.91973084	0.89583352	0.94362815
TDFa	177	Rarefaction	0	39.5025279	35.6920316	43.3130241	0.925251	0.90105399	0.94944801
TDFa	189	Rarefaction	0	40.3714779	36.384068	44.3588877	0.93027133	0.90578291	0.95475976
TDFa	201	Rarefaction	0	41.1823578	37.0124497	45.3522659	0.93491377	0.9101328	0.95969475

TDFa	213	Rarefaction	0	41.9392523	37.5812504	46.2972543	0.93925234	0.91416173	0.96434294
TDFa	214	Observed	0	42	37.6260726	46.3739274	0.93960134	0.91448421	0.96471846
TDFa	215	Extrapolation	0	42.0603987	37.6705073	46.45029	0.93994833	0.91480513	0.96509153
TDFa	226	Extrapolation	0	42.7023153	38.134286	47.2703446	0.94363618	0.91824006	0.96903231
TDFa	237	Extrapolation	0	43.304811	38.5540871	48.055535	0.94709756	0.92152018	0.97267493
TDFa	248	Extrapolation	0	43.8703067	38.9325905	48.8080229	0.95034637	0.92466517	0.97602756
TDFa	259	Extrapolation	0	44.4010745	39.2724685	49.5296805	0.95339566	0.9276872	0.97910412
TDFa	271	Extrapolation	0	44.9429895	39.6022871	50.283692	0.956509	0.93085267	0.98216532
TDFa	282	Extrapolation	0	45.4078825	39.8698395	50.9459255	0.95917984	0.93363944	0.98472023
TDFa	293	Extrapolation	0	45.8442258	40.1066697	51.5817819	0.96168665	0.93631998	0.98705333
TDFa	304	Extrapolation	0	46.2537727	40.3151371	52.1924082	0.96403953	0.93889716	0.98918189
TDFa	315	Extrapolation	0	46.6381688	40.4974788	52.7788587	0.96624791	0.94137349	0.99112232
TDFa	327	Extrapolation	0	47.0306379	40.6690734	53.3922025	0.96850267	0.94396277	0.99304256
TDFa	338	Extrapolation	0	47.3673257	40.8034426	53.9312087	0.97043696	0.94623607	0.99463784
TDFa	349	Extrapolation	0	47.683337	40.9177726	54.4489014	0.97225246	0.94841611	0.99608882
TDFa	360	Extrapolation	0	47.9799417	41.0137659	54.9461175	0.97395648	0.95050547	0.99740749
TDFa	371	Extrapolation	0	48.2583315	41.093001	55.4236619	0.97555584	0.95250684	0.99860485
TDFa	383	Extrapolation	0	48.542568	41.1620402	55.9230957	0.9771888	0.95459307	0.99978453
TDFa	394	Extrapolation	0	48.7864061	41.210825	56.3619873	0.97858967	0.95641948	1
TDFa	405	Extrapolation	0	49.0152699	41.2470144	56.7835254	0.9799045	0.95816663	1
TDFa	416	Extrapolation	0	49.2300788	41.2717469	57.1884107	0.98113859	0.95983739	1
TDFa	428	Extrapolation	0	49.4493991	41.2868907	57.6119076	0.9823986	0.96157631	1
TDFb	1	Rarefaction	0	1	1	1	0.09367529	0.07235841	0.11499217
TDFb	12	Rarefaction	0	8.11325789	7.63076264	8.59575314	0.53883771	0.49181306	0.58586235
TDFb	24	Rarefaction	0	12.8320497	11.8695943	13.7945052	0.66397212	0.62517619	0.70276805
TDFb	36	Rarefaction	0	16.526753	15.170093	17.8834129	0.71955653	0.68522367	0.7538894
TDFb	48	Rarefaction	0	19.6856741	17.9835215	21.3878267	0.75496037	0.72303964	0.78688111
TDFb	60	Rarefaction	0	22.4719169	20.4542393	24.4895946	0.78192467	0.75170281	0.81214653
TDFb	72	Rarefaction	0	24.9630651	22.6517164	27.2744137	0.80409367	0.7752454	0.83294194
TDFb	83	Rarefaction	0	27.0288286	24.464092	29.5935652	0.82150068	0.79369537	0.849306
TDFb	95	Rarefaction	0	29.0774057	26.2509203	31.9038911	0.83808352	0.81119961	0.86496743
TDFb	107	Rarefaction	0	30.9384095	27.8630753	34.0137437	0.85266718	0.82649434	0.87884002
TDFb	119	Rarefaction	0	32.6337349	29.3200526	35.9474171	0.8656083	0.83995504	0.89126156
TDFb	131	Rarefaction	0	34.1816393	30.6377911	37.7254875	0.87715595	0.85185204	0.90245985
TDFb	143	Rarefaction	0	35.5977911	31.8297556	39.3658266	0.88749224	0.86238932	0.91259517
TDFb	154	Rarefaction	0	36.7919435	32.8218951	40.761992	0.89602398	0.87099256	0.9210554
TDFb	166	Rarefaction	0	37.9926225	33.8042367	42.1810084	0.90441113	0.87935115	0.92947111

TDFb	178	Rarefaction	0	39.0974899	34.691212	43.5037679	0.91193665	0.88674974	0.93712357
TDFb	190	Rarefaction	0	40.1163728	35.4910758	44.7416697	0.91869233	0.89328769	0.94409696
TDFb	202	Rarefaction	0	41.0580339	36.2111603	45.9049076	0.92476368	0.89905152	0.95047585
TDFb	214	Rarefaction	0	41.9302326	36.8579002	47.0025649	0.93023256	0.90411657	0.95634854
TDFb	215	Observed	0	42	36.9086644	47.0913356	0.93066456	0.90451173	0.95681738
TDFb	216	Extrapolation	0	42.0693355	36.9589611	47.1797098	0.93109388	0.90490502	0.95728273
TDFb	227	Extrapolation	0	42.8042669	37.4822202	48.1263135	0.93564454	0.90911564	0.96217343
TDFb	238	Extrapolation	0	43.4906624	37.953165	49.0281598	0.93989466	0.91313331	0.96665602
TDFb	249	Extrapolation	0	44.1317274	38.3755532	49.8879015	0.94386411	0.91697684	0.97075137
TDFb	261	Extrapolation	0	44.7828841	38.785203	50.7805652	0.94789604	0.92098513	0.97480694
TDFb	272	Extrapolation	0	45.3386089	39.117553	51.5596647	0.95133706	0.92449882	0.97817529
TDFb	283	Extrapolation	0	45.8576328	39.4119895	52.3032762	0.95455083	0.92786514	0.98123651
TDFb	294	Extrapolation	0	46.3423797	39.6716004	53.013159	0.95755236	0.93108911	0.9840156
TDFb	306	Extrapolation	0	46.8347575	39.9184988	53.7510162	0.96060113	0.93444914	0.98675313
TDFb	317	Extrapolation	0	47.2549736	40.1144501	54.3954972	0.96320309	0.9373899	0.98901628
TDFb	328	Extrapolation	0	47.6474381	40.2839432	55.010933	0.96563321	0.94020186	0.99106456
TDFb	339	Extrapolation	0	48.0139837	40.4293116	55.5986557	0.96790284	0.94288929	0.99291639
TDFb	351	Extrapolation	0	48.3862994	40.562916	56.2096828	0.9702082	0.94568407	0.99473234
TDFb	362	Extrapolation	0	48.7040495	40.6646321	56.743467	0.9721757	0.94812504	0.99622635
TDFb	373	Extrapolation	0	49.000815	40.7483827	57.2532472	0.97401325	0.95045481	0.9975717
TDFb	384	Extrapolation	0	49.2779816	40.8158507	57.7401124	0.97572946	0.95267767	0.99878124
TDFb	396	Extrapolation	0	49.5595114	40.8726972	58.2463256	0.97747268	0.95498567	0.99995968
TDFb	407	Extrapolation	0	49.7997809	40.9109728	58.6885889	0.97896041	0.95699866	1
TDFb	418	Extrapolation	0	50.0241826	40.9373525	59.1110128	0.9803499	0.95891768	1
TDFb	430	Extrapolation	0	50.2521169	40.9539546	59.5502793	0.98176125	0.96090871	1
LTF	1	Rarefaction	0	1	1	1	0.06403765	0.05268305	0.07539225
LTF	19	Rarefaction	0	12.1935421	11.5997258	12.7873584	0.59266387	0.55795737	0.62737037
LTF	38	Rarefaction	0	18.2269321	17.1042051	19.3496591	0.75551098	0.72971693	0.78130504
LTF	57	Rarefaction	0	22.1428436	20.6452897	23.6403975	0.82808465	0.80792368	0.84824561
LTF	76	Rarefaction	0	25.0138116	23.236886	26.7907373	0.86848922	0.85149755	0.88548088
LTF	95	Rarefaction	0	27.2631454	25.2650104	29.2612805	0.89444209	0.87909618	0.909788
LTF	114	Rarefaction	0	29.0949327	26.9102191	31.2796463	0.91274879	0.89828701	0.92721057
LTF	133	Rarefaction	0	30.6236923	28.2728805	32.9745041	0.92643739	0.91252633	0.94034845
LTF	152	Rarefaction	0	31.9219038	29.4172286	34.426579	0.93703972	0.92353608	0.95054335
LTF	171	Rarefaction	0	33.0396337	30.3885409	35.6907266	0.94543205	0.93226604	0.95859807
LTF	190	Rarefaction	0	34.0134955	31.2205899	36.8064011	0.9521722	0.9392975	0.9650469
LTF	209	Rarefaction	0	34.8712202	31.9393526	37.8030878	0.95764366	0.94501601	0.97027132

LTF	228	Rarefaction	0	35.634291	32.565158	38.703424	0.96212266	0.94969015	0.97455517
LTF	247	Rarefaction	0	36.3196336	33.1140931	39.5251742	0.96581323	0.9535125	0.97811397
LTF	266	Rarefaction	0	36.940784	33.5989948	40.2825732	0.96886825	0.95662423	0.98111228
LTF	285	Rarefaction	0	37.5087267	34.0301774	40.9872759	0.97140337	0.95913145	0.98367529
LTF	304	Rarefaction	0	38.0325081	34.4159792	41.6490371	0.9735069	0.96111631	0.9858975
LTF	323	Rarefaction	0	38.5196883	34.7631765	42.2762001	0.97524694	0.96264403	0.98784986
LTF	342	Rarefaction	0	38.9766764	35.0773043	42.8760484	0.97667639	0.96376671	0.98958606
LTF	343	Observed	0	39	35.0930113	42.9069887	0.97674438	0.96381629	0.98967247
LTF	344	Extrapolation	0	39.0232556	35.1086395	42.9378717	0.97681218	0.96386578	0.98975859
LTF	362	Extrapolation	0	39.4304521	35.3765119	43.4843923	0.97799935	0.96474444	0.99125425
LTF	380	Extrapolation	0	39.8168011	35.6192024	44.0143998	0.97912573	0.96561193	0.99263952
LTF	398	Extrapolation	0	40.18337	35.837344	44.5293959	0.98019444	0.96647636	0.99391252
LTF	416	Extrapolation	0	40.5311714	36.0318223	45.0305204	0.98120844	0.9673392	0.99507768
LTF	434	Extrapolation	0	40.8611661	36.2037062	45.518626	0.98217052	0.96819895	0.9961421
LTF	452	Extrapolation	0	41.1742659	36.3541847	45.9943472	0.98308335	0.96905294	0.99711376
LTF	470	Extrapolation	0	41.4713357	36.4845134	46.4581581	0.98394944	0.96989827	0.99800062
LTF	488	Extrapolation	0	41.7531963	36.595972	46.9104205	0.9847712	0.97073219	0.99881021
LTF	506	Extrapolation	0	42.0206262	36.6898326	47.3514198	0.98555087	0.97155229	0.99954946
LTF	524	Extrapolation	0	42.2743644	36.7673359	47.7813928	0.98629064	0.97235656	1
LTF	542	Extrapolation	0	42.5151117	36.8296759	48.2005476	0.98699252	0.97314339	1
LTF	560	Extrapolation	0	42.7435334	36.8779894	48.6090774	0.98765848	0.97391153	1
LTF	578	Extrapolation	0	42.9602604	36.9133504	49.0071704	0.98829033	0.97466003	1
LTF	596	Extrapolation	0	43.1658915	36.9367674	49.3950156	0.98888984	0.97538822	1
LTF	614	Extrapolation	0	43.3609948	36.9491833	49.7728062	0.98945865	0.97609569	1
LTF	632	Extrapolation	0	43.5461092	36.9514765	50.1407419	0.98999835	0.97678218	1
LTF	650	Extrapolation	0	43.7217462	36.9444634	50.4990289	0.99051041	0.97744763	1
LTF	668	Extrapolation	0	43.888391	36.9289015	50.8478805	0.99099625	0.97809211	1
LTF	686	Extrapolation	0	44.046504	36.9054922	51.1875157	0.99145722	0.97871579	1
OF.SMS	1	Rarefaction	0	1	1	1	0.07128398	0.06164625	0.08092171
OF.SMS	26	Rarefaction	0	13.8757351	13.1925612	14.558909	0.71313206	0.68828947	0.73797465
OF.SMS	51	Rarefaction	0	19.2312239	18.09519	20.3672577	0.84056091	0.82197145	0.85915037
OF.SMS	76	Rarefaction	0	22.5260675	21.051427	24.000708	0.89172021	0.87550066	0.90793977
OF.SMS	101	Rarefaction	0	24.8794745	23.1074524	26.6514965	0.9186074	0.90404876	0.93316604
OF.SMS	127	Rarefaction	0	26.7684645	24.7148753	28.8220537	0.93545944	0.92226131	0.94865758
OF.SMS	152	Rarefaction	0	28.2442718	25.9418149	30.5467287	0.94627421	0.93411673	0.95843168
OF.SMS	177	Rarefaction	0	29.4887246	26.9559383	32.0215109	0.95409895	0.94276509	0.96543282
OF.SMS	202	Rarefaction	0	30.561758	27.8146424	33.3088737	0.9600437	0.94935279	0.97073461

OF.SMS	228	Rarefaction	0	31.5377168	28.5821449	34.4932888	0.96487764	0.95469338	0.97506189
OF.SMS	253	Rarefaction	0	32.3695448	29.2252018	35.5138877	0.96859514	0.95876321	0.97842708
OF.SMS	278	Rarefaction	0	33.1170219	29.7933596	36.4406842	0.97162848	0.96203326	0.9812237
OF.SMS	303	Rarefaction	0	33.7953151	30.2999887	37.2906416	0.97412958	0.96467028	0.98358887
OF.SMS	328	Rarefaction	0	34.4163637	30.7553727	38.0773547	0.97620627	0.96679604	0.9856165
OF.SMS	354	Rarefaction	0	35.0118434	31.1832802	38.8404066	0.97800051	0.96856291	0.98743811
OF.SMS	379	Rarefaction	0	35.5440756	31.5574534	39.5306979	0.97943505	0.96990736	0.98896274
OF.SMS	404	Rarefaction	0	36.0434037	31.9003851	40.1864223	0.98062967	0.97095881	0.99030052
OF.SMS	429	Rarefaction	0	36.5153994	32.2163983	40.8144005	0.98161834	0.97175851	0.99147817
OF.SMS	455	Rarefaction	0	36.9824561	32.5203981	41.4445142	0.98245614	0.97235672	0.99255556
OF.SMS	456	Observed	0	37	32.5316364	41.4683636	0.98248501	0.97237574	0.99259428
OF.SMS	457	Extrapolation	0	37.017515	32.5428426	41.4921873	0.98251384	0.97239485	0.99263282
OF.SMS	480	Extrapolation	0	37.4124997	32.7917651	42.0332342	0.98316384	0.97285104	0.99347663
OF.SMS	504	Extrapolation	0	37.8090122	33.0335812	42.5844431	0.98381635	0.97334359	0.99428912
OF.SMS	528	Extrapolation	0	38.1901571	33.2572744	43.1230398	0.98444358	0.97383885	0.99504831
OF.SMS	552	Extrapolation	0	38.55653	33.4631138	43.6499462	0.9850465	0.97433192	0.99576108
OF.SMS	576	Extrapolation	0	38.9087035	33.6514609	44.1659461	0.98562605	0.97482163	0.99643047
OF.SMS	600	Extrapolation	0	39.2472279	33.8227663	44.6716894	0.98618314	0.97530786	0.99705841
OF.SMS	624	Extrapolation	0	39.5726321	33.9775595	45.1677047	0.98671864	0.97579061	0.99764666
OF.SMS	648	Extrapolation	0	39.8854247	34.1164327	45.6544166	0.98723338	0.97626967	0.99819709
OF.SMS	672	Extrapolation	0	40.1860944	34.240025	46.1321638	0.98772817	0.97674468	0.99871167
OF.SMS	696	Extrapolation	0	40.4751111	34.3490067	46.6012156	0.98820379	0.97721514	0.99919244
OF.SMS	720	Extrapolation	0	40.7529265	34.4440652	47.0617877	0.98866097	0.97768049	0.99964146
OF.SMS	744	Extrapolation	0	41.0199746	34.5258939	47.5140552	0.98910044	0.97814016	1
OF.SMS	768	Extrapolation	0	41.2766728	34.5951824	47.9581631	0.98952287	0.9785936	1
OF.SMS	792	Extrapolation	0	41.5234221	34.6526089	48.3942353	0.98992893	0.9790403	1
OF.SMS	816	Extrapolation	0	41.7606082	34.6988348	48.8223817	0.99031925	0.97947979	1
OF.SMS	840	Extrapolation	0	41.9886018	34.7345003	49.2427033	0.99069445	0.97991169	1
OF.SMS	864	Extrapolation	0	42.207759	34.7602216	49.6552965	0.9910551	0.98033564	1
OF.SMS	888	Extrapolation	0	42.4184224	34.7765888	50.0602561	0.99140178	0.98075139	1
OF.SMS	912	Extrapolation	0	42.6209212	34.7841651	50.4576773	0.99173502	0.98115871	1
RS	1	Rarefaction	0	1	1	1	0.10289021	0.08951145	0.11626898
RS	45	Rarefaction	0	15.5911976	14.8740942	16.3083009	0.86230373	0.84977559	0.87483188
RS	89	Rarefaction	0	19.7052412	18.6521881	20.7582944	0.93605037	0.92689327	0.94520747
RS	133	Rarefaction	0	21.9446676	20.6342185	23.2551167	0.95912598	0.95192807	0.96632389
RS	177	Rarefaction	0	23.4939193	21.9739059	25.0139328	0.96959732	0.96358011	0.97561452
RS	221	Rarefaction	0	24.6896093	22.9934951	26.3857235	0.97574416	0.97048052	0.9810078

RS	265	Rarefaction	0	25.6619849	23.8145573	27.5094125	0.97991531	0.97515591	0.98467471
RS	309	Rarefaction	0	26.4769348	24.4972051	28.4566645	0.98296516	0.97854775	0.98738257
RS	353	Rarefaction	0	27.1742037	25.0768377	29.2715697	0.98529667	0.98111235	0.989481
RS	397	Rarefaction	0	27.7800449	25.5762443	29.9838454	0.98713951	0.98311738	0.99116164
RS	441	Rarefaction	0	28.312622	26.0108342	30.6144098	0.98863827	0.98473306	0.99254348
RS	485	Rarefaction	0	28.7848928	26.3914237	31.1783619	0.98988777	0.98606889	0.99370664
RS	529	Rarefaction	0	29.2063801	26.725896	31.6868642	0.9909508	0.98719437	0.99470723
RS	573	Rarefaction	0	29.5843343	27.0202348	32.1484338	0.99186878	0.98815286	0.99558469
RS	617	Rarefaction	0	29.9245137	27.279182	32.5698453	0.99266874	0.98897114	0.99636634
RS	661	Rarefaction	0	30.2317066	27.5066563	32.956757	0.9933682	0.98966579	0.99707061
RS	705	Rarefaction	0	30.510071	27.7060128	33.3141293	0.99397865	0.99024741	0.99770989
RS	749	Rarefaction	0	30.7633445	27.8801959	33.6464932	0.99450798	0.99072313	0.99829282
RS	793	Rarefaction	0	30.9949622	28.0318154	33.9581091	0.99496222	0.99109813	0.9988263
RS	794	Observed	0	31	28.0350177	33.9649824	0.99497173	0.99110598	0.99883748
RS	795	Extrapolation	0	31.0050283	28.0382096	33.971847	0.99498122	0.99111382	0.99884862
RS	836	Extrapolation	0	31.2032157	28.1602746	34.2461568	0.9953554	0.99143642	0.99927437
RS	878	Extrapolation	0	31.3909257	28.2680995	34.5137519	0.99570979	0.99176564	0.99965394
RS	920	Extrapolation	0	31.564313	28.3594595	34.7691666	0.99603714	0.99208867	0.99998561
RS	961	Extrapolation	0	31.7208032	28.433742	35.0078643	0.99633259	0.99239481	1
RS	1003	Extrapolation	0	31.8690202	28.4956035	35.2424369	0.99661242	0.99269717	1
RS	1045	Extrapolation	0	32.0059279	28.5441133	35.4677426	0.9968709	0.99298727	1
RS	1087	Extrapolation	0	32.1323894	28.5803198	35.684459	0.99710966	0.99326485	1
RS	1128	Extrapolation	0	32.2465267	28.6047737	35.8882796	0.99732515	0.99352378	1
RS	1170	Extrapolation	0	32.3546299	28.6196302	36.0896297	0.99752924	0.99377694	1
RS	1212	Extrapolation	0	32.4544847	28.6250971	36.2838724	0.99771777	0.99401822	1
RS	1254	Extrapolation	0	32.5467204	28.6220562	36.4713845	0.99789191	0.99424803	1
RS	1295	Extrapolation	0	32.6299673	28.6116727	36.648262	0.99804907	0.99446172	1
RS	1337	Extrapolation	0	32.7088133	28.5941949	36.8234317	0.99819793	0.99467016	1
RS	1379	Extrapolation	0	32.7816432	28.5705022	36.9927842	0.99833543	0.99486847	1
RS	1421	Extrapolation	0	32.848916	28.541254	37.156578	0.99846244	0.99505711	1
RS	1462	Extrapolation	0	32.9096328	28.5079241	37.3113414	0.99857708	0.99523237	1
RS	1504	Extrapolation	0	32.9671397	28.4694325	37.4648469	0.99868565	0.99540322	1
RS	1546	Extrapolation	0	33.0202588	28.4270452	37.6134724	0.99878594	0.99556572	1
RS	1588	Extrapolation	0	33.0693247	28.3812275	37.757422	0.99887857	0.99572028	1
