# Supplementary material for: Diversity of Plant Communities Surrounding the Hot Springs on the Eastern Flank of the Sierra Madre Oriental, Northeastern Mexico
Source: Biology (Basel). 2025 Apr 7;14(4):382. doi: 10.3390/biology14040382 (PMC12025227; doi:10.3390/biology14040382)
Supplement: Supplementary file 1 [file biology-14-00382-s001.zip › Table S3.pdf]

Table S3. Indicator species by vegetation type around hot springs on the eastern flank of the Sierra Madre Oriental, northeastern Mexico and combination of those by IndVal result. Signif. codes: 0 = ***, 0.001 = **, 0.01 = *

Specie	TDFb	OF.SMS	LTF	RS	TDFa	IndVal	Sign
Guazuma ulmifolia Lam.	+					1	**
Sabal mexicana Mart	+					1	**
Trichilia havanensis Jacq.	+					1	**
Piscidia piscipula Sarg.	+					0.949	*
Esenbeckia runyonii C. V. Morton		+				0.987	*
Abutilon fruticosum Guill. & Perr.			+			1	*
Callaeum macropterum (Moc. & Sesse ex DC.) D.M. Johnson			+			1	*
Cordia boissieri A. DC.			+			1	*
Havardia pallens Britton & Rose			+			1	*
Justicia pilosella Hilsenb.			+			0.956	*
Croton cortesianus Kunt			+			0.922	*
Karwinskia humboldtiana (Schult.) Zucc.			+			0.922	*
Agave lechuguilla Torr.				+		1	**
Agave striata Zucc.				+		1	**
Astrolepis integerrima (Hook.) D.M. Benham & Windham				+		1	**
Dasylirion berlandieri S. Watson				+		1	**
Dodonaea viscosa (L.) Jacq.				+		1	**
Echinocactus platyacanthus Link & Otto.				+		1	**
Euphorbia antisyphilitica Zucc.				+		1	**
Hechtia glomerata Zucc.				+		1	**
Opuntia rastrera Engelm.				+		1	**
Pellaea ovata Weath.				+		1	**
Ocotea tampicensis Hemsl.					+	1	*

Lysiloma divaricatum J.F.Macbr.					+	0.965	*
Capparidastrum frondosum (Jacq.) Cornejo & Iltis					+	0.894	*
Pisonia aculeata L.	+				+	1	***
Acalypha schiedeana Schltdl.	+				+	0.913	*
Bauhinia divaricata L.	+				+	0.913	*
Gochnatia hypoleuca (DC.) A. Gray		+		+		0.913	*
Helietta parvifolia A. Gray (Benth).		+	+	+		0.943	*
